# Liposomal Encapsulated Rhodomyrtone: A Novel Antiacne Drug

**DOI:** 10.1155/2013/157635

**Published:** 2013-05-16

**Authors:** Julalak Chorachoo, Thanaporn Amnuaikit, Supayang P. Voravuthikunchai

**Affiliations:** ^1^Natural Products Research Center and Department of Microbiology, Faculty of Science, Prince of Songkla University, Hat Yai 90112, Thailand; ^2^Department of Pharmaceutical Technology, Faculty of Pharmaceutical Sciences, Prince of Songkla University, Hat Yai 90112, Thailand

## Abstract

Rhodomyrtone isolated from the leaves of *Rhodomyrtus tomentosa* possesses antibacterial, anti-inflammatory, and anti-oxidant activities. Since rhodomyrtone is insoluble in water, it is rather difficult to get to the target sites in human body. Liposome exhibited ability to entrap both hydrophilic and hydrophobic compounds and easily penetrate to the target site. The present study aimed to develop a novel liposomal encapsulated rhodomyrtone formulations. In addition, characterization of liposome, stability profiles, and their antiacne activity were performed. Three different formulations of total lipid concentrations 60, 80, and 100 **μ**mol/mL were used. Formulation with 60 **μ**mol/mL total lipid (phosphatidylcholine from soybean and cholesterol from lanolin in 4 : 1, w/w) exhibited the highest rhodomyrtone encapsulation efficacy (65.47 ± 1.7%), average particle size (209.56 ± 4.8 nm), and **ζ**-potential (–41.19 ± 1.3 mV). All formulations demonstrated good stability when stored for 2 months in dark at 4°C as well as room temperature. Minimal inhibitory concentration and minimal bactericidal concentration values of liposomal formulation against 11 clinical bacterial isolates and reference strains ranged from 1 to 4 and from 4 to 64 **μ**g/mL, respectively, while those of rhodomyrtone were 0.25–1 and 0.5–2 **μ**g/mL, respectively. The MIC and MBC values of liposome formulation were more effective than topical drugs against *Staphylococcus aureus* and *Staphylococcus epidermidis*.

## 1. Introduction 

Acne vulgaris is a common human skin disease that affects 80% of young people worldwide who aged between 11 to 30 years [1]. Pathogenesis of acne lesion formation is multifactorial including initial colonization by *Propionibacterium acne*, *Staphylococcus epidermidis*, and *Staphylococcus aureus*, followed by lipolytic enzyme production, sebum excretion, comedogenesis, certain inflammatory mediators involving sebaceous hyperplasia, follicular hyperkeratinization, hormone imbalance, and immune hypersensitivity [2–4]. In addition, free radicals generated by environmental factors such as toxins and UV rays of sunlight can attack and damage the vital components of healthy skin cells, leading to skin problems such as wrinkles, scars, age spots, and acne lesion. 

 Numerous treatments including topical application [5, 6], oral administration of antibiotics [7], oral contraceptives [8, 9], and antioxidants [10] can improve control of mild to moderate acne. Inevitably, the use of antibiotics has resulted in increase in antibiotic resistant acne-inducing bacterial strains [11]. Recently, oral isotretinoin has been claimed to be the most effective therapy and its use begins in severe disease cases. However, its use is limited by teratogenicity and several side effects [6]. Therefore, therapeutic agents, predominantly from natural products, may provide an appropriate approach to control these problems. 

 Antimicrobial and antioxidant properties of various phytochemicals isolated from Thai medicinal plants have been reported [11, 12]. As a part of our on-going research in developing a topical applicative medication to alleviate skin infections, rhodomyrtone isolated from the leaves of *Rhodomyrtus tomentosa* (Aiton) Hassk. (Family Myrtaceae) was of interest to us. Crude extract from the leaves and rhodomyrtone have been reported to possess both very strong antibacterial activity [13–15] and antioxidant property [16]. In spite of its potent biological activity, rhodomyrtone is highly insoluble in water and difficult to get to target sites. 

The use of liposomes as drug carriers has been well documented [17, 18]. There are several reports indicating the advantages of the use of liposomal substance as carriers. Liposomes are composed of lipids similar to those present in biological membranes; therefore, they are expected to be biocompatible, biodegradable, practically nonimmunogenic, and nontoxic [19, 20]. In addition, liposomes are well suited for delivery of therapeutic agents because they usually provide a sustained drug releasing their content slowly, gradually, and increase overall drug efficacy. Therefore, the objectives of this study were (i) to develop novel liposomal encapsulated rhodomyrtone formulations, (ii) to characterize their stability profiles, and (iii) to investigate their antiacne activities.

## 2. Material and Methods

### 2.1. Rhodomyrtone

Rhodomyrtone, a pure compound from the leaves of *Rhodomyrtus tomentosa*, was isolated according to the previously described method [21]. 

### 2.2. Preparation of Liposomal Encapsulated Rhodomyrtone

Liposomal encapsulated rhodomyrtone was prepared by modified ethanol injection method [22]. Rhodomyrtone was dissolved in absolute ethanol (Merck, Germany) to get concentration of 100 mg/mL. Lipid phase was prepared as 60, 80, and 100 *μ*mol/mL total with lipid concentration by dissolving phosphatidylcholine from soybean (Sigma, USA) and cholesterol from lanolin (Fluka, Japan) with ratio of 4 : 1 in 10 mL ethanol. 10 *μ*L of rhodomyrtone was added in the above lipid suspension followed by sonication for 30 min. 10 mL of sonicate MilliQ water and lipid phase was warmed in water bath separately till the temperature of both phase reached 60°C. Water phase was mixed with lipid phase and the dispersion mixture was continually mixed for five minutes. The mixture was poured into a round bottle flask connected to a rotary evaporator (Eyela Rotary Vacuum Evaporator N-100 series, Tokyo, Japan) to get rid of ethanol. Subsequently, liposomal encapsulated rhodomyrtone was transferred into a glass bottle, sealed, and stored till further use.

#### 2.2.1. Size Measurement and *ζ*-Potential

Size and *ζ*-potential of liposomal samples were determined by *ζ*-potential analyzer (Zeta PALS, Brookhaven). 10 *μ*L of each liposomal suspension was taken and suitably diluted in distilled water, and *z*-average mean and *ζ*-potential were measured. All measurements were made for three times at 25°C. 

#### 2.2.2. Scanning Electron Microscope Observations

Morphological and particle sizes of prepared liposomal encapsulated rhodomyrtone formulations have been investigated by SEM (Quanta 400, FEI, Czech Republic). Briefly, 200 *μ*L of liposome formulation was diluted with 3 mL MilliQ water. A drop of diluted liposome was allowed to dry on the cover slip and then stained with crystal violet solution for 1 min. Excess dye was rinsed out with water followed by fixing with Gram's iodine solution for 1 min [23]. The sample was then coated with gold in a sputter coater under an argon atmosphere (50 Pa) at 50 mA for 50 seconds and investigated under SEM at 30,000X magnification.

#### 2.2.3. Encapsulation Efficacy

Encapsulation efficiency was determined as the percentage of rhodomyrtone encapsulated in liposome to the original amount of rhodomyrtone added. To determine drug release efficiency of liposome, lipid vesicles were lysed using 100% Triton X-100 (Baker analyzed, USA). Concisely, 100 *μ*L of liposomal suspension was added to 100 *μ*L of 100% Triton X-100 and vortexed for 5 min to ease lysis of the liposomal encapsulated rhodomyrtone. Free rhodomyrtone was separated from liposome by centrifugation using ultracentrifuge (optima L-100 xp, USA) at 60,000 rpm, 4°C for 2 h. Concentrations of the drug in the filtrate, total drug, and free drug were quantitatively analyzed using HPLC with UV-VIS detection. The encapsulation efficacy was calculated using the following formula:

(1) EE  (%)=[(T−FT)]×100,

where EE is encapsulation efficacy, *T* is total rhodomyrtone for encapsulation, and *F* is free drug in sample.

### 2.3. Quantitative Determination of Active Substances

Quantitative determination of active substances was performed using HPLC Agilent 1100 liquid chromatographic system which consists of an Agilent Chemstation for GC, LC, LC/MSD, CE, UV-VIS detector (Agilent 1100, USA), and A/D Systems-Rev. 08. Ox. An aliquot of 20 *μ*L of each sample solution was directly injected into the HPLC system with a mixture of acetonitrile (LB Science Co., Thailand) and water (85 : 15, v/v) as the mobile phase at flow rate of 1.0 mL/min. Separation was performed in Symmetry C8 analytical column (4.6 × 150 mm, particle size, 3 *μ*m) at 30°C and the peak was detected at 254 nm. The column was equilibrated for 6 min before every subsequent run and was saturated with the mobile phase for at least 3 h before each assay. Rhodomyrtone was used as the internal standard for the assay.

### 2.4. Method Validation

Analytical validation for rhodomyrtone derived from liposomal preparation was assessed for specificity, linearity, accuracy, precision, limit of detection (LOD), and limit of quantification (LOQ) [24].

#### 2.4.1. Specificity

From HPLC chromatograms, specificity of liposomal encapsulated rhodomyrtone was compared with standard rhodomyrtone. The resolution of the neighbouring peaks, tailing factor, and number of theoretical plates was determined. 

#### 2.4.2. Linearity and Range

Linearity of the method was evaluated by processing six calibration curves of rhodomyrtone. Standard solutions containing 1, 5, 10, 20, 40, and 60 *μ*g/mL of rhodomyrtone were prepared and analyzed in triplicate using HPLC. Peak area ratios between rhodomyrtone and internal standard were plotted against different concentrations of rhodomyrtone. A linear least squares regression analysis was conducted to determine slope, intercept, and coefficient of determination (*r* ^2^). 

#### 2.4.3. Precision

Both intraday and interday precision experiments were conducted. Standard solution of rhodomyrtone was used to achieve repeatability testing. Data of repeatability was from three separate injections within the same day. Data used to calculate percent recovery of standard (%RSD) of interday precision was the content of three samples analyzed from three different days (three injections daily).

#### 2.4.4. Accuracy

Accuracy of the method was measured through recovery test of the drug spiked at different concentration levels (low, medium, and high) in the samples. Standard rhodomyrtone stock solution containing 100 mg/mL was added in increments at five different concentrations (5, 30, 200, 500, and 1,000 *μ*g/mL) to spike the sample solutions. The solutions were filtered through 0.45 *μ*m nylon membrane filter and analyzed by HPLC in triplicate.

#### 2.4.5. Limit of Detection (LOD) and Limit of Quantification (LOQ)

LOD and LOQ were calculated based on the standard deviation of the regression line or the standard deviation of *y*-intercepts of regression lines and slope. LOD and LOQ were calculated using the following formula:

(2) LOD=(3.3×SD)S,  LOQ=(10×SD)S,

where SD is standard deviation of *y*-intercepts of regression lines and *S* is slope of standard curve. 

### 2.5. Bacterial Strains and Media

Clinical isolates of multidrug resistant *Staphylococcus aureus* NPRC 302, 308, 317, and 322; *Staphylococcus epidermidis* NPRC 529, 537, 573, and 577; and *Propionibacterium acnes *NPRC 021, 036, and 039 were obtained from the culture collection at Natural Products Research Center, Prince of Songkla University. Reference strains *S. aureus* ATCC 25923,* S. epidermidis* ATCC 35984, and *P. acne* DMST 14916 were included as controls. *S. aureus* and *S. epidermidis* were cultured on Mueller-Hinton agar (MHA) (Difco, France) at 35°C for 24 h. Mueller-Hinton broth (MHB) (Difco, France) was used for testing antibacterial activity. *P. acne *was cultured in brain heart infusion (BHI) broth (Difco, France) supplemented with 1% glucose and 0.5% yeast extract. The bacteria were cultured in an anaerobic atmosphere using BBL GasPak systems (LB Science, Thailand).

#### 2.5.1. Determination of Minimal Inhibitory Concentrations (MICs) and Minimal Bactericidal Concentrations (MBCs)

A modified microdilution method [25] was used to determine the MIC of liposomal encapsulated rhodomyrtone. Aliquots (50 *μ*L) of liposomal encapsulated rhodomyrtone and rhodomyrtone were diluted in a 96-well microtitre plate to yield final concentrations of 0.25–128 *μ*g/mL, and 50 *μ*L of MHB was added. One hundred microliter of each bacterial isolate containing approximately 10^6^ CFU/mL was incubated at 35°C for 15 h. After incubation, resazurin (Sigma, Germany), a redox indicator, was added to each well, incubated at 35°C for 3 h (*S. aureus* and *S. epidermidis*), and incubated anaerobically at 35°C for 3 days (*P. acne*). MICs were recorded at the lowest concentration of the agent that completely inhibited the bacterial growth from at least 2 out of 3 well. MBC, the lowest concentration of liposomal encapsulated rhodomyrtone completely preventing bacterial growth, was determined with liposomal encapsulated rhodomyrtone that gave MICs by subculturing on fresh MHA (*S. aureus* and *S. epidermidis*) and BHI agar supplemented with 1% glucose and 0.5% yeast extract (*P. acne*).

## 3. Results 

### 3.1. Method Development 

#### 3.1.1. Specificity

**Figure 1:**
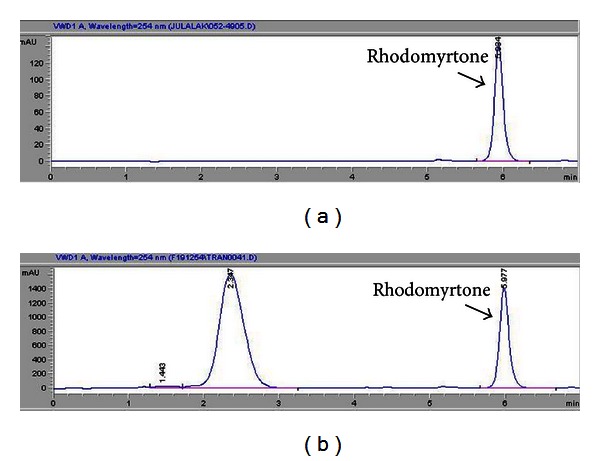
HPLC chromatogram of specificity validation for HPLC analysis of rhodomyrtone after derivatization: (a) pure compound rhodomyrtone (60 *μ*g/mL) and (b) liposomal encapsulated rhodomyrtone (1,000 *μ*g/mL).

HPLC chromatograms obtained are shown in Figure 1. From the chromatograms, it was observed that the peak of standard rhodomyrtone and liposomal encapsulated rhodomyrtone sample appeared at the same retention time, about 6 min. No interference of unwanted peaks was observed at the retention time of rhodomyrtone indicating the method is specific.

#### 3.1.2. Linearity and Range

Peak of rhodomyrtone appeared after 6 min from the start of HPLC. The calibration curve of rhodomyrtone quantitation was linear at the concentrations that ranged from 1 to 60 *μ*g/mL. Linear regression equation for rhodomyrtone was *y* = 23.3804*x* − 7.6887. The coefficient of determination (*r* ^2^) was greater than 0.999 representing high degree of linearity. 

#### 3.1.3. Precision and Accuracy

Intra- and interday variability was utilized to determine the precision of this newly developed method. It was found that %RSD ranged from 0.68 to 0.96% during intraday and 0.67 to 1.28% during interday precision suggesting that the method is precise (Table 1). Accuracy of the method was measured through recovery test of the drug spiked at high, medium, and low concentrations of the calibration curve. The average percent recovery of rhodomyrtone ranged from 95 to 109%, proving that the method is highly accurate (Table 2). 

#### 3.1.4. Limit of Detection (LOD) and Limit of Quantification (LOQ)

LODs and LOQs of the current method were 0.004 and 0.013 *μ*g/mL, respectively, indicating that the method was sufficiently sensitive to be used for the drug entrapment study.

### 3.2. Formulation and Characterization of Liposomal Encapsulated Rhodomyrtone

Characterization of three liposomal formulations with different concentration of lipid prepared by modified ethanol injection method is presented in Table 3. 

#### 3.2.1. Size Measurement and *ζ*-Potential

Range of particle sizes was between 200 and 460 nm. The formulation with lipid 60 *μ*mol/mL exhibited the smallest particle size of 209.56 ± 4.8 nm, demonstrating electrical polarity and particle distribution. Surface charge of 60, 80, and 100 *μ*mol/mL liposome formulations was −41.19, −46.29, and −50.34 mV, respectively. 

#### 3.2.2. SEM

**Figure 2:**
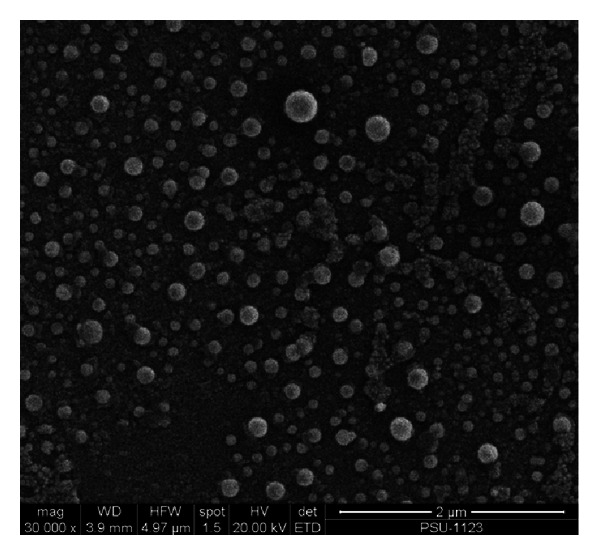
Scanning electron micrograph of liposome encapsulated rhodomyrtone formulation. The magnification is 30,000X.

All liposomal formulations possess fine sphere shape with relatively monodispersed distributed size (Figure 2). 

#### 3.2.3. Entrapment Efficacy

Entrapment efficacy (EE) was calculated as the percentage of liposomal encapsulated rhodomyrtone. Encapsulation rate of rhodomyrtone content was between 51% and 65% of the targeted concentration (1 mg/mL) in the prototype formulation due to loss of compound during the formulation process. Liposome with total lipid at 60 *μ*mol/mL exhibited greater %EE (65.47 ± 1.7) followed by 100 *μ*mol/mL (63.05 ± 0.8) and 80 *μ*mol/mL (53.60 ± 1.2) of total lipids containing liposomes.

#### 3.2.4. Physical Appearance

Physical appearance of all liposome formulations (total amount of lipid 60, 80, and 100 *μ*mol/mL) stored at 4°C and 25°C for 2 months was evaluated. At the end of the 2nd month, all the liposomal formulations stored at 4°C were stable but slight changes in the colour and viscosity of the liposomal suspension were observed stored at 25°C. No significant changes in %EE were observed in all liposomal formulations. In contrast, significant changes in particle size and *ζ*-potential were noticed in all liposome formulations stored at 4°C and 25°C. 

### 3.3. MICs and MBCs

MIC and MBC values of liposomal encapsulated rhodomyrtone against 11 clinical isolates and reference strains ranged from 1 to 4 and from 4 to 64 *μ*g/mL, respectively, while those of rhodomyrtone were 0.25–1 and 0.5–2 *μ*g/mL, respectively (Table 4). Interestingly, liposome formulation was more effective than azelaic acid, benzoyl peroxide, and clindamycin against *Staphylococcus aureus* ATCC 25923 and *Staphylococcus epidermidis* ATCC 35984 (Table 5). 

## 4. Discussion

Incorporation of compounds in liposome is known to enhance the therapeutic index of antimicrobial drugs, either by increasing the drug concentration in bacterial cells or by decreasing the exposure in normal host tissues. Phosphatidylcholine is a main lipid constituent of several commercially available liposome products due to neutral charge and its nontoxic biodegradable profile. Liposomes dispersed in aqueous solution generally face physical and chemical instabilities after long-term storage. Hydrolysis and oxidation of phospholipids and liposome aggregation are the common cause of liposome instabilities [26]. The incorporation of cholesterol within phosphatidylcholine liposomes enhances liposome stability, modulates rigidity, and reduces serum induced stability [27]. However, a balanced concentration of cholesterol is essential to form stable liposomes to prevent leakage, while excess or deficit of cholesterol could result in destabilization of lipid bilayer membrane leading to decreased drug entrapment and increased leakage [28].

In view of the above advantage of liposome mediated delivery systems, we have encapsulated rhodomyrtone in liposome formulations with total lipids content of 60, 80, and 100 *μ*mol/mL by modified ethanol injection methods. Rhodomyrtone was encapsulated in liposome by physical entrapment in lipid phase of liposome. There were no chemical reactions between liposome constituents and drug as revealed by appearance of the peak in HPLC at the same retention time for liposome encapsulated rhodomyrtone and free rhodomyrtone. The method was specific since the recovery of the drug was almost 100%. The particle size of higher lipid content liposome was slightly higher than the liposome with lower lipid content. The size of particles increased during storage which may be due to aggregation or fusion of liposomes. The negative charge of liposome formulation was due to the presence of cholesterol on the surface of liposome. The charge on the liposome depends on the head group composition of lipid and pH of the solution. The bigger sized liposome was found more negatively charged, compared with smaller liposome, because the tendency of zeta potential increased when total lipid content increased [28]. The amount of charge on the surface of liposome influences stability, kinetics, biodistribution, and interactions with the targeted cells. Higher charges on particles produce strong repulsive force between same charge particles that keep the particles away from each other and make the particles more stable. Negatively charged liposomes are less prone to aggregation and comparatively more stable in suspension [29]. During storage the charge on liposome decreased due to aggregation of liposomal particles and decrease in total surface area. 

In general, the EE of lipid soluble drugs is higher at higher lipid concentration. However, in the present study, we did not observe any significant difference in EE between 60, 80, and 100 *μ*mol/mL total lipid content liposome. During storage at different temperatures also the EE was stable even after 2 months which may be because of the ratio of phosphatidylcholine and cholesterol that made the liposome stable and prevented leakage during storage. There was no visible aggregation of particles observed with storage as precipitate was not found in the liposomal suspension. There were no effects of storage temperature on particle size, zeta potential, and EE when liposomes were stored at 4 and 25°C. Similar trends of change in size, zeta potential, and EE were observed when liposome was stored at both temperatures. However, slight change in colour and viscosity was observed in the liposomes stored at 25°C. 

 Although the MIC and MBC values of liposomal encapsulated rhodomyrtone were acceptable, they were comparatively higher than the free rhodomyrtone. This could be due to slow release of rhodomyrtone from liposome into the bacterial cells. Free rhodomyrtone can interact with bacterial cells directly and act faster compared with liposomal encapsulated rhodomyrtone. In contrast, higher efficacy of liposomal encapsulated drugs was reported, compared with free drugs [30, 31]. Moreover, enhanced antibacterial activity of liposomal encapsulated amikacin against * E. coli*, *S. faecalis*, and* S. aureus* was observed whereas reduced activity against *P. aeruginosa* was observed [32]. In general, liposomes fuse with bacterial outer membrane and release their contents into the cells [30]. Drugs which face physical barrier in penetration to bacterial cells could be improved by forming liposomal formulations that increase the concentration of drug inside bacterial cells for the drugs. Since the present liposomal formulation releases drugs slowly, higher drug dose can be applied for slow and controlled releases of drug at the target site and make the host cells less prone to side effects of drug.

## 5. Conclusion

A simple and inexpensive simultaneous HPLC method for the measurement of rhodomyrtone employing UV-VIS detection was developed and successful in analysis of liposomal encapsulated rhodomyrtone samples. The present study demonstrated potent antimicrobial activity of liposomal encapsulated rhodomyrtone which may be useful in the treatment of acne vulgaris. Although the antibacterial effects of liposomal encapsulated rhodomyrtone against *P. acnes*,* S. aureus*, and* S. epidermidis* were demonstrated, their mechanism remains unknown. Further research is needed to evaluate skin permeability and clinical trials of liposomal encapsulated rhodomyrtone.

## Figures and Tables

**Table 1:** Intra- and interday precision study.

Rhodomyrtone (*μ*g/mL)	Intraday				Interday	
	Content (*μ*g/mL)			RSD (%)	Content (*μ*g/mL)	RSD (%)
	Day 1	Day 2	Day 3			
5	4.97 ± 0.61	4.97 ± 0.61	4.96 ± 0.28	0.96	4.90 ± 0.45	1.28
30	27.71 ± 2.47	28.20 ± 2.47	28.89 ± 1.67	0.90	29.94 ± 2.28	0.99
60	57.87 ± 3.70	58.15 ± 3.70	59.05 ± 2.34	0.68	58.36 ± 3.04	0.67

The results are the mean of three independent experiments ± S.E.

**Table 2:** Results of accuracy determination by recovery study.

Spiked concentration (*μ*g/mL)	Rhodomyrtone	
	Recovery (%)	RSD (%)
5	98.05 ± 0.45	1.28
30	100.72 ± 1.02	0.44
200	108.91 ± 3.42	0.28
500	102.23 ± 3.33	0.13
1,000	95.78 ± 4.92	0.09

The results are the mean of three independent experiments ± S.E.

**Table 3:** Stability of different liposomal encapsulated rhodomyrtone formulations.

Total lipid (*µ*mol/mL)	Test (month)	Temp. (°C)	Particle size (nm)	*ζ*-potential (mV)	Entrapment efficacy (%)	Physical appearance
60	0	25	209.56 ± 4.8	−41.19 ± 1.3	65.47 ± 1.7	Milky suspension
	2	4	217.72 ± 4.1	−34.62 ± 1.3	64.57 ± 1.9	Milky suspension
		25	220.76 ± 4.9	−35.98 ± 2.4	61.88 ± 1.1	Yellow color of milky suspension
	4	4	261.24 ± 7.7	−34.78 ± 1.5	64.94 ± 3.2	Milky suspension
		25	264.10 ± 6.7	−34.36 ± 1.2	61.73 ± 2.8	Yellow color of milky suspension
	6	4	262.60 ± 9.3	−34.91 ± 2.2	65.49 ± 2.4	Milky suspension
		25	283.26 ± 8.3	−35.96 ± 1.6	57.06 ± 3.1	Slightly viscous of yellow milky suspension

80	0	25	262.24 ± 5.6	−46.29 ± 1.2	53.60 ± 1.2	Milky suspension
	2	4	419.21 ± 1.7	−32.36 ± 1.1	51.87 ± 1.4	Milky suspension
		25	421.63 ± 0.9	−38.10 ± 1.6	46.66 ± 1.2	Slightly viscous of yellow milky suspension

100	0	25	274.64 ± 0.7	−50.34 ± 1.3	63.05 ± 0.8	Milky suspension
	2	4	438.71 ± 1.3	−45.54 ± 1.5	60.91 ± 1.5	Slightly viscous of yellow milky suspension
		25	459.43 ± 1.4	−41.96 ± 1.8	60.57 ± 0.9	Slightly viscous of yellow milky suspension

The results are the mean of three independent experiments ± S.E.

**Table 4:** Comparison of minimum inhibitory concentrations (MICs) and minimum bactericidal concentrations (MBCs) of liposomal encapsulated rhodomyrtone formulation at different time against *Propionibacterium acnes*, *Staphylococcus aureus*, and *Staphylococcus epidermidis*.

Isolates	MICs/MBCs (*μ*g/mL)			
	Rhodomyrtone	Liposomal formulation		
		Before	After 6 months	
			4°C	25°C
*Propionibacterium acnes *				
NPRC 021	0.25/0.5	1/32	ND	ND
NPRC 036	0.25/1	2/32	ND	ND
NPRC 039	0.25/1	1/8	ND	ND
DMST 14916	0.25/1	1/16	ND	ND
*Staphylococcus aureus *				
NPRC 302	1/2	1/32	1/32	1/64
NPRC 308	0.5/1	1/8	1/8	1/32
NPRC 317	0.25/1	2/32	2/64	32/128
NPRC 322	1/2	1/16	1/32	1/64
ATCC 25923	0.25/1	1/4	1/4	1/8
*Staphylococcus epidermidis *				
NPRC 529	0.5/1	2/64	4/64	128/128
NPRC 537	0.5/2	2/32	2/32	64/64
NPRC 573	0.25/2	4/32	4/32	32/64
NPRC 577	0.25/1	2/32	2/32	4/64
ATCC 35984	0.25/2	2/8	2/8	4/32

ND: not done.

**Table 5:** Comparison of minimum inhibitory concentrations (MICs) and minimum bactericidal concentrations (MBCs) of topical drugs and liposomal encapsulated rhodomyrtone against *Staphylococcus aureus* ATCC 25923 and *Staphylococcus epidermidis* ATCC 35984.

Topical drugs	MICs/MBCs (*µ*g/mL)	
	*Staphylococcus aureus *	*Staphylococcus epidermidis *
	ATCC 25923	ATCC 35984
Azelaic acid (20% w/w)	256/512	64/128
Benzoyl peroxide (2.5% w/w)	>1024	>1024
Clindamycin (1% w/v)	1/64	>1024
Liposomal encapsulated rhodomyrtone	1/4	2/8
